# Rapid Classification of Sugarcane Nodes and Internodes Using Near-Infrared Spectroscopy and Machine Learning Techniques

**DOI:** 10.3390/s24227102

**Published:** 2024-11-05

**Authors:** Siramet Veerasakulwat, Agustami Sitorus, Vasu Udompetaikul

**Affiliations:** 1 Department of Agricultural Engineering, School of Engineering, King Mongkut’s Institute of Technology Ladkrabang, Bangkok 10520, Thailand; siramet.ve@kmitl.ac.th; 2 Research Center for Artificial Intelligence and Cyber Security, National Research and Innovation Agency (BRIN), Bandung 40135, Indonesia; agustami.sitorus@brin.go.id

**Keywords:** sugarcane, node and internode classification, NIR spectroscopy, spectral preprocessing, machine learning, precision agriculture

## Abstract

Accurate and rapid discrimination between nodes and internodes in sugarcane is vital for automating planting processes, particularly for minimizing bud damage and optimizing planting material quality. This study investigates the potential of visible-shortwave near-infrared (Vis–SWNIR) spectroscopy (400–1000 nm) combined with machine learning for this classification task. Spectral data were acquired from the sugarcane cultivar Khon Kaen 3 at multiple orientations, and various preprocessing techniques were employed to enhance spectral features. Three machine learning algorithms, linear discriminant analysis (LDA), K-Nearest Neighbors (KNNs), and artificial neural networks (ANNs), were evaluated for their classification performance. The results demonstrated high accuracy across all models, with ANN coupled with derivative preprocessing achieving an F1-score of 0.93 on both calibration and validation datasets, and 0.92 on an independent test set. This study underscores the feasibility of Vis–SWNIR spectroscopy and machine learning for rapid and precise node/internode classification, paving the way for automation in sugarcane billet preparation and other precision agriculture applications.

## 1. Introduction

The escalating global demand for sugar and bioenergy has driven the expansion of sugarcane cultivation. Worldwide sugarcane production is projected to reach 1.92 billion tons in 2023/24, marking a 3.4% increase from the previous year [1]. However, the industry is grappling with significant challenges, including labor shortages, rising production costs, and the need for sustainable practices that minimize environmental impact.

The planting phase of sugarcane cultivation is particularly crucial, as it directly influences crop establishment and subsequent yield. The quality of planting material, particularly the presence of healthy and undamaged buds, is critical for successful crop establishment [2]. Several planting techniques are currently employed, each with its advantages and limitations.

Mechanized planting methods, such as vertical sugarcane planting and billet planting, offer increased efficiency and uniformity compared to traditional manual planting. The progress in mechanization has been notable, particularly in regions like Thailand, where the adoption of vertical planters and billet planters has increased significantly [3].

Vertical planters utilize whole sugarcane stalks, which are fed into the machine and then cut into billets of appropriate length, typically containing two or three buds, for planting [3]. The cutting process in vertical planters, while faster, can sometimes lead to bud damage, affecting the planting material’s quality.

Billet planters, on the other hand, use pre-cut sugarcane billets, often obtained from the harvesting process. The billet planter feeds these billets into its hopper and then plants them directly into the soil. However, the billets sourced from harvesters often suffer significant bud damage due to the mechanical stresses involved in harvesting, impacting their viability for planting [2]. Studies have shown that bud damage due to the harvesting process can be as high as 35.59% [2].

Another emerging technique is bud chip seedling planting, which involves the direct planting of pregerminated sugarcane buds [4]. This method significantly reduces the amount of planting material required and has shown promising results in terms of seedling survival rate and yield improvement [5,6]. However, it necessitates precise cutting of sugarcane buds to ensure their viability and successful germination. The current manual cutting process can be time-consuming and prone to errors, highlighting the need for automation. The development of specialized seedling transplanters further underscores the importance of precision and efficiency in bud chip seedling planting [7].

The common challenge across these planting techniques is the potential for bud damage, which can negatively impact crop establishment and yield. The development of a precision planter that can accurately identify and cut sugarcane stalks at the nodes, minimizing bud damage, is therefore crucial for improving planting efficiency and overall sugarcane productivity.

Near-infrared spectroscopy (NIRS) has emerged as a powerful non-destructive tool for analyzing the chemical composition and physical properties of agricultural products [8]. The interaction of near-infrared light with organic molecules, such as carbohydrates, proteins, and water, generates unique spectral signatures that can be used to develop classification models. Recent advancements in NIRS technology, including miniaturization, portability, and hyperspectral imaging, have expanded its applications in agriculture, enabling rapid, infield analysis and real time monitoring [9].

The integration of NIRS with machine learning algorithms has shown great promise in various agricultural applications, including fruit quality assessment [10], crop disease detection [11], and soil analysis [12].

Recent advancements in NIRS technology have led to the development of portable and rapid detection tools for various applications, including citrus quality analysis [13], plant leaf analysis [14], food quality analysis [15], and milk quality analysis [16]. This trend highlights the growing potential of NIRS for real-time, in-field analysis in agriculture and other industries.

In the context of sugarcane, NIRS has been employed for tasks such as predicting sugar content [17], fiber content [18], and disease detection [19]. However, its application for node and internode classification remains relatively unexplored, particularly in the visible-shortwave near-infrared (Vis–SWNIR) range (400–1000 nm).

This study addresses this gap by pioneering the use of Vis–SWNIR spectroscopy combined with machine learning for rapid and precise node/internode classification of sugarcane stalks. The specific objectives are as follows: (1) To evaluate the feasibility of Vis–SWNIR spectroscopy for classifying sugarcane nodes and internodes. (2) To develop and compare the performance of different machine learning models (LDA, KNN, and ANN) for this classification task. (3) To investigate the impact of spectral preprocessing techniques on model accuracy and robustness.

The successful implementation of this technology could pave the way for automation in sugarcane billet preparation and bud chip seedling production, leading to reduced bud damage, improved planting efficiency, and enhanced overall productivity in the sugarcane industry. Furthermore, this research contributes to the broader field of precision agriculture by demonstrating the potential of NIRS and machine learning for rapid and non-destructive classification tasks in crop production systems.

## 2. Materials and Methods

### 2.1. Sugarcane Sample Collection and Preparation

**Figure 1:**
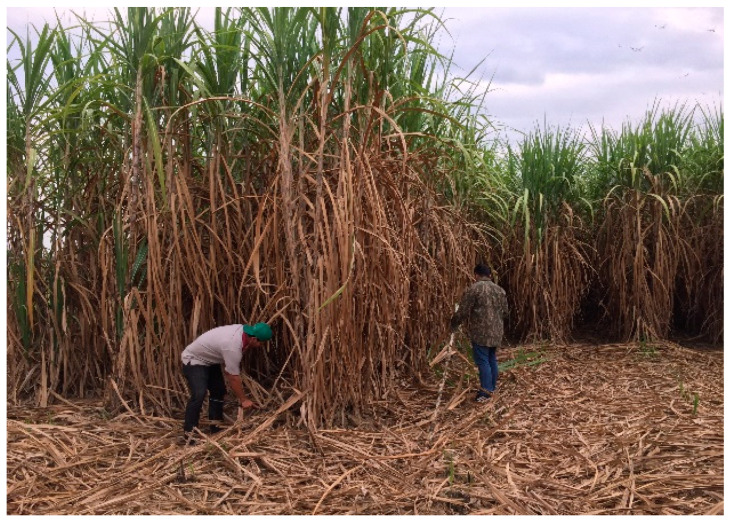
Field sampling of sugarcane stalks for spectral analysis.

Fifty-five sugarcane stalks of the Khon Kaen 3 variety were collected from two fields (twenty-five and thirty stalks, respectively) (Figure 1). The stalks were at a maturity stage of 10 months, suitable for planting, and were sourced from fields specifically prepared for seed cane production. For each stalk, one node was randomly selected from each of the following positions:

1. Upper: fifth node from the top.
2. Middle: Approximately the middle node based on stalk length.
3. Bottom: fifth node from the bottom.

The internode selected for scanning was the one directly below each chosen node.

### 2.2. Vis–SWNIR Spectroscopy Data Acquisition

Spectral data collection was performed using a Vis–SWNIR spectrometer (AvaSpec–2048–USB2, Avantes, Apeldoorn, The Netherlands) equipped with a linear array CCD sensor (2048 pixels), offering a spectral resolution of 0.6 nm. The integration time for spectral acquisition was set to 5 ms. A tungsten halogen lamp (AvaLight–HAL–S, Avantes, Apeldoorn, The Netherlands) served as the light source, covering both the visible and near-infrared regions (350 to 2500 nm).

**Figure 2:**
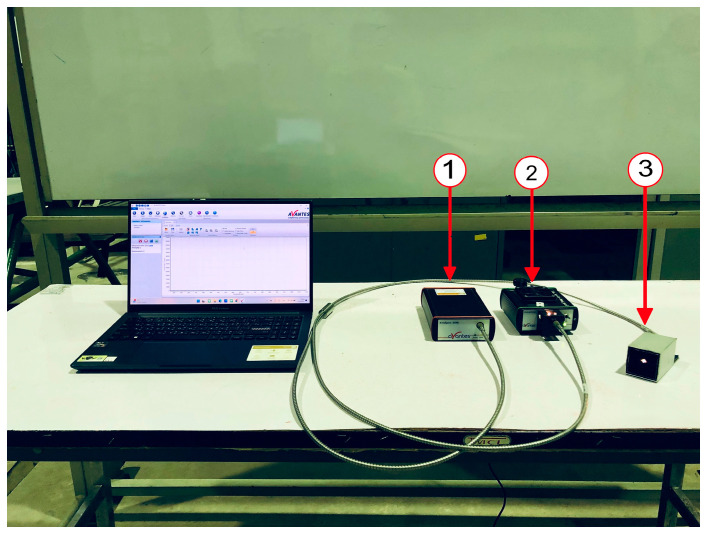
Experimental setup for Vis–SWNIR spectral data acquisition from sugarcane stalks: (1) spectrometer, (2) light source, and (3) probe.

A fiber optic reflectance probe (FCR–7IR200–2–BX, Avantes, Apeldoorn, The Netherlands) with seven 200-micrometer-diameter optical fibers (six for illumination and one for collection) was used for spectral acquisition. The probe has a 0.22 numerical aperture (NA) and a 2 m length. The probe was encased in custom-built aluminum housing (5 cm × 5 cm × 10 cm) filled with black PU foam to prevent light leakage. The probe was placed in direct contact with the sugarcane sample during scanning. The experiment took place in a laboratory room with a temperature of 25 ± 2 °C and a relative humidity of 50% to 60%. The equipment and setup are shown in Figure 2.

**Figure 3:**
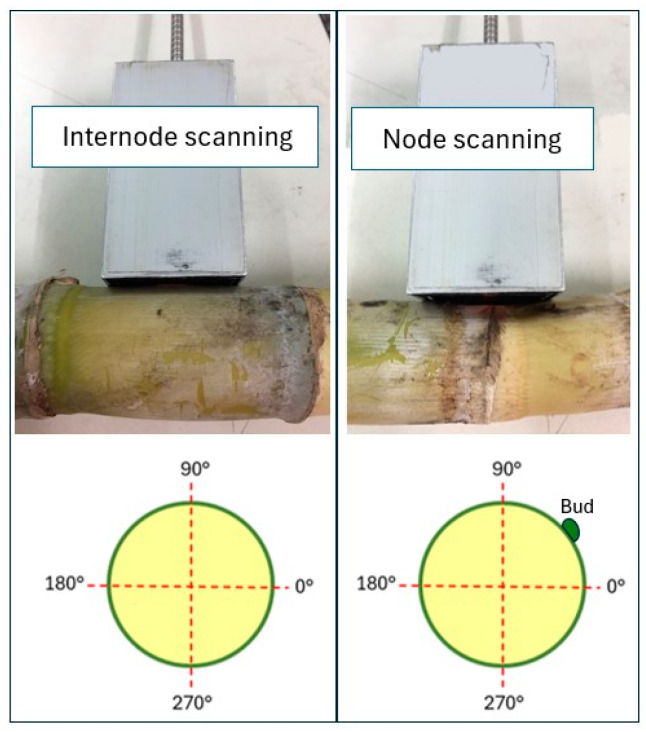
Schematic representation of the node/internode scanning angles for spectral data acquisition.

For each node and internode, the following scans were performed (Figure 3):

- Internode: Four scans were taken around the middle of the internode from four perpendicular directions. The scanning direction was adjusted by rotating the stalk and maintaining the contact between the stalk surface and the probe end.
- Node: Five scans were taken: (a) four scans around the selected node (excluding the bud) from four perpendicular directions and (b) one scan directly at the bud.

### 2.3. Data Preprocessing and Classification Modeling

Raw spectral data often contain noise from various sources, including instrument variations, baseline shifts, and light scattering [20]. Preprocessing these spectra is essential to remove noise and enhance the relevant spectral features for model development [21]. However, the optimal preprocessing method can vary depending on the specific dataset and its characteristics [22].

In this study, we applied six common spectral preprocessing techniques to the raw Vis–SWNIR spectra, including mean normalization (MN), Norm_L2, infinity norm (Norm_inf), standard normal variate (SNV), multiplicative scatter correction (MSC) and derivative (DL). MN adjusts each spectrum by subtracting its mean value to center it around zero. Norm_L2 rescales the spectrum to a Euclidean length (norm) of 1, while Norm_inf scales the spectrum by dividing all values by the maximum absolute value. SNV corrects for scatter effects by centering and scaling each spectrum based on its standard deviation. On the other hand, MSC reduces scattering effects by regressing each spectrum against the mean spectrum and correcting for both additive and multiplicative offsets. Lastly, the DL calculates the first derivative of the spectrum using the Savitzky–Golay filter, which emphasizes changes in slope and highlights subtle spectral features.

After the preprocessing stage, this study constructed classification models using three machine learning algorithms, including linear discriminant analysis (LDA), K-nearest neighbors (KNNs), and artificial neural networks (ANNs). These algorithms were purposely selected to represent linear and non-linear classification approaches, thereby enabling a comprehensive assessment of their suitability for this specific task.

#### 2.3.1. Linear Discriminant Analysis (LDA)

**Figure 4:**
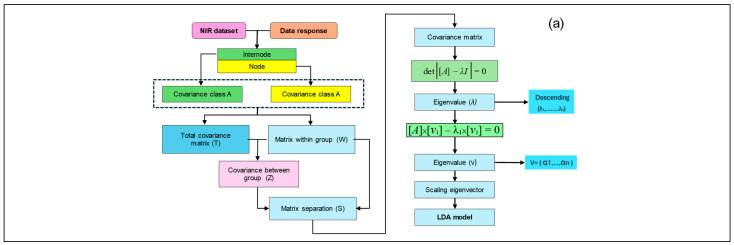

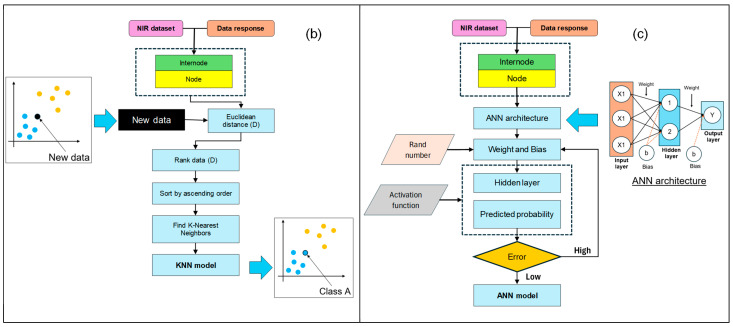
Schematic representation of machine learning algorithms used in this study. (**a**) Linear Discriminant Analysis (LDA), (**b**) k-Nearest Neighbors (KNN) and (**c**) Artificial Neural Network (ANN). In these diagrams, rectangles represent datasets, calculations, and models. Arrows indicate the flow of data.

LDA is a dimensionality reduction and classification technique that projects high-dimensional data onto a lower-dimensional space while maximizing the separation between classes [23]. This approach is beneficial for spectral data, which often exhibit high dimensionality and potential collinearity. LDA can improve classification accuracy and reduce computational complexity by identifying the linear combinations of features that best discriminate between classes. In LDA, the dimensionality reduction process works by projecting data from a high-dimensional space onto a lower-dimensional subspace that maximizes class separation. It achieves this by finding linear combinations of the original features that best differentiate the classes. The process begins by calculating the mean vectors for each class and the overall mean of the data. LDA then computes the between-class and within-class scatter matrices, optimizing for the linear transformation that maximizes the ratio of between-class and within-class variance. Refer to Figure 4a for a visual depiction of an LDA. This approach ensures that the transformed space retains the most relevant information for classification.

#### 2.3.2. K-Nearest Neighbors (KNN)

KNNs is a non-parametric, instance-based learning algorithm that classifies new data points based on their similarity to the K–nearest neighbors in the training set [24,25]. The class of a new sample is determined through majority voting among its neighbors. KNNs is appreciated for its simplicity, interpretability, and ability to handle non-linear relationships. To implement KNN, the algorithm calculates the distance (typically Euclidean) between the new data point and all points in the training set. The K closest points (neighbors) are identified based on these distances. The new data point is assigned to the class that is most common among these neighbors, with each neighbor having an equal vote. In ties, the algorithm may choose the nearest neighbor’s class or use other strategies like weighted voting, where closer neighbors have a higher influence (Figure 4b). This method ensures flexibility and adaptability in classifying complex, non-linear data patterns.

#### 2.3.3. Artificial Neural Network (ANN)

ANNs are computational models inspired by the human brain, which are capable of learning complex patterns and relationships in data [26]. See Figure 4c for a graphical representation of an ANN. They consist of interconnected nodes (neurons) organized in layers, with each connection having an associated weight. ANNs can model non-linear relationships, making them suitable for complex classification tasks in which linear models may not easily define the decision boundaries. To implement the ANNs, we employed the scikit-learn library, which provides a flexible framework for building and optimizing neural networks. The architecture and hyperparameters were tuned extensively through a grid search to achieve optimal classification performance. Specifically, we explored various configurations of hidden layers and neuron counts, including single, double, and triple hidden layers with sizes ranging from (4), (4, 4), (4, 4, 4) to (256, 256, 256), as shown in Table 1. This range of configurations allowed us to identify the architecture that best captures the complex, non-linear patterns present in the spectral data.

Additionally, we tested four different activation functions (Table 1) to evaluate their impact on model performance. The “ReLU” (Rectified Linear Unit) function was particularly effective due to its ability to efficiently address the vanishing gradient problem and model non-linear relationships. Other activation functions, such as “tanh” and “logistic”, were also evaluated to compare their suitability for this specific classification task. For other hyperparameters, we used the default settings of the scikit-learn library. The solver was set to Adam, an adaptive moment estimation optimizer known for its efficiency in handling non-stationary objectives and large datasets. We applied an L2 regularization term of 0.0001 to prevent overfitting by penalizing large weights, while the learning rate was kept constant with an initial value of 0.001. The model was trained for a maximum of 200 iterations, and early stopping was not employed to allow the model to train until convergence or until the maximum iteration count was reached.

#### 2.3.4. Hyperparameter Tuning

The performance of machine learning models is influenced by their hyperparameters. To optimize the classification accuracy, we performed hyperparameter tuning for each algorithm using grid search. The hyperparameters considered and their tuning ranges are listed in Table 1. For the LDA, we tuned the number of component parameters within the range of 1 to 20. This range was selected to provide sufficient flexibility for the model to determine the optimal number of components that would maximize class separation while minimizing complexity. It aligns with standard practices in LDA applications, ensuring that the model captures the essential variability in the data without overfitting. Next, we optimized the number of neighbor parameters for the KNN within the range of 1 to 20. This range was selected to compromise sensitivity and stability: lower K values may render the model too sensitive to noise, while higher values may smooth the decision boundaries too much. By examining this range, according to established KNN modeling protocols, we sought to determine the best K that ensures precise classification and resilience. Finally, for ANN, we tuned the hidden layer sizes with configurations ranging from (4) to (256, 256, 256) to explore shallow and deep architectures. This range allowed us to identify the optimal network complexity needed to capture the non-linear patterns in the spectral data. Additionally, we tested four activation functions (identity, logistic, tanh, and relu) to evaluate their effectiveness in enhancing model performance. The tuning ranges and choices were based on best our practices to ensure that the network architecture was flexible enough to adapt to the specific characteristics of the dataset. The optimal hyperparameters were selected based on the model’s performance on a validation set, using the F1-score as the primary evaluation metric. The F1-score was chosen because it provides a balanced assessment of both precision and recall, making it particularly suitable for evaluating models on datasets with class imbalances or when both false positives and false negatives are of significant concern.

### 2.4. Model Evaluation

The performance of the classification algorithms was assessed using a confusion matrix [27] and common classification metrics, including accuracy, precision, recall, and the F1-score [28] (Table 2). The F1-score, which is the harmonic mean of precision and recall, was particularly emphasized, as it provides a balanced measure of the model’s ability to correctly identify both nodes and internodes.

From a total of 495 scans, 50 scans were randomly selected for external unknown validation, and the remaining 445 scans were used for calibration with a random state of 62. The calibration set was further divided into 80% for training and 20% for internal validation, using a random state of 62, and optimized by the GridsearchCV method by 5-fold cross-validation (5f–CV) in accuracy metrics. This study utilized Python version 3.11.4 along with the Scikit-learn machine learning library version 1.2.2, executed on the Jupyter Notebook platform version 6.5.4 within the Anaconda environment.

**Figure 5:**
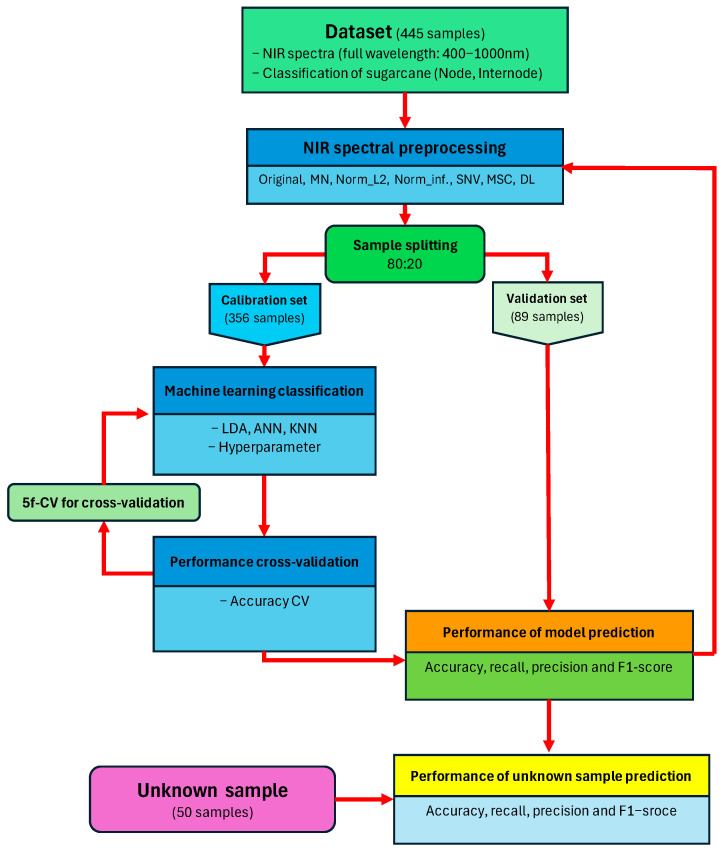
Overview of the node/internode classification model development process.

The overall workflow for developing and evaluating the node/internode classification models is illustrated in Figure 5.

## 3. Results

### 3.1. NIR Spectra of Sugarcane Samples

**Figure 6:**
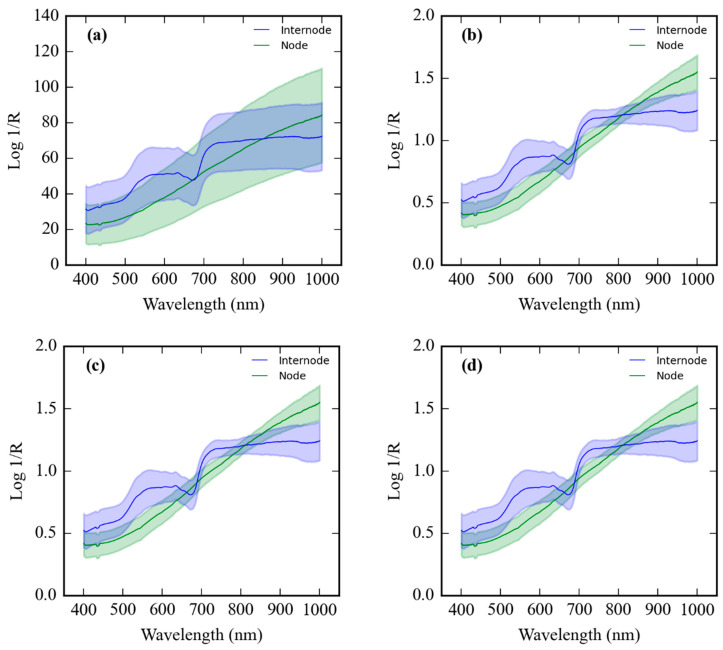

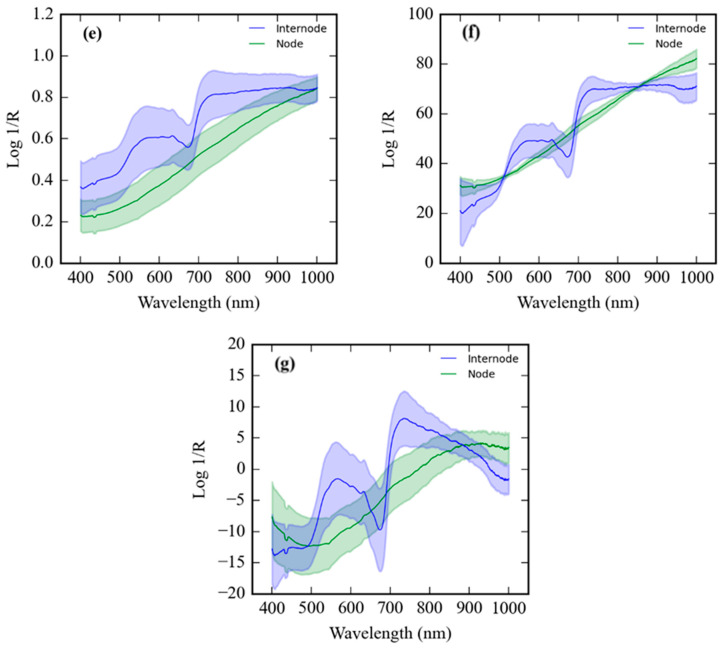
Average Vis–SWNIR spectra of sugarcane nodes and internodes with ±1 standard deviation: (**a**) original, (**b**) MN, (**c**) Norm_L2, (**d**) Norm_inf, (**e**) MSC, (**f**) SNV, and (**g**) DL.

Figure 6a–g present the spectra of node and internode regions of sugarcane for various preprocessing methods: original, mean normalization (MN), L2_Norm (Norm_L2), infinity norm (Norm_inf), multiplicative scatter correction (MSC), standard normal variate (SNV), and derivative (DL), respectively.

The raw Vis-SWNIR spectra of sugarcane nodes and internodes exhibit subtle differences in absorbance intensities. The derivative transformation enhances these differences, revealing distinct spectral patterns for each class. For instance, the prominent peaks around 650–700 nm, more distinct in internodes, might be associated with chlorophyll or other pigments, which are more abundant in the photosynthetically active internodes. The more pronounced peaks and valleys in the derivative spectra of the nodes could be related to their varied textures and the presence of buds.

### 3.2. Classification Model Performance

**Figure 7:**
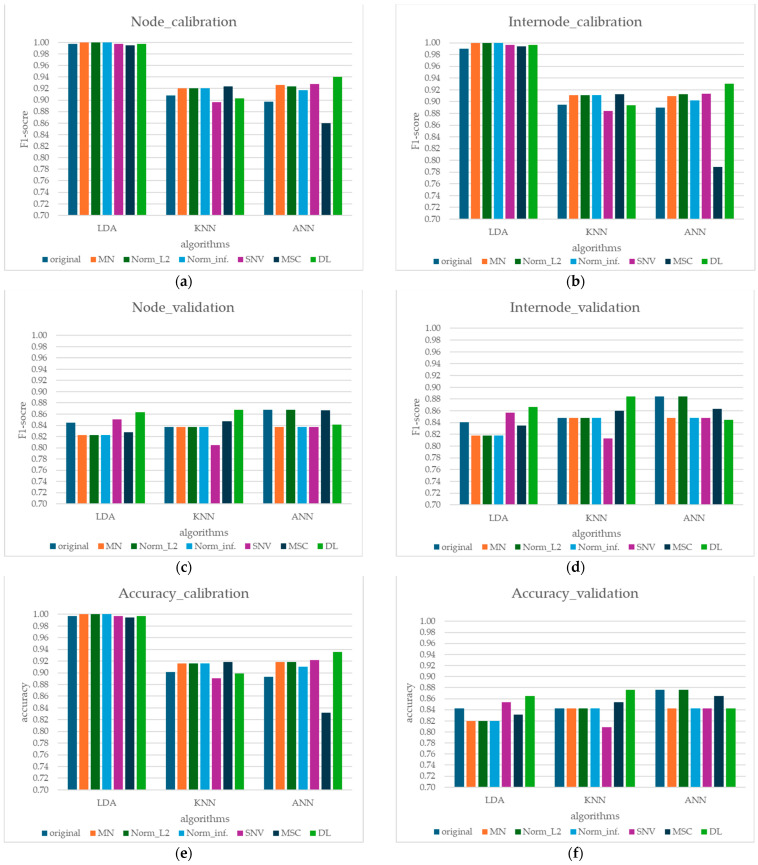
Comparison of performance metrics of calibration and validation models for different preprocessing methods and machine learning algorithms: (**a**) node F1-score (calibration), (**b**) internode F1-score (calibration), (**c**) node F1-score (validation), (**d**) internode F1-score (validation), (**e**) model accuracy (calibration), and (**f**) model accuracy (validation).

Table 3 and Figure 7a–f presents the performance of the calibration and validation datasets for models built using three algorithms: LDA, KNN, and ANN. A total of 445 data points were used for calibration, consisting of 356 training data points and 89 for internal validation. Each algorithm was constructed using seven sets of spectra: one raw spectrum and six preprocessed spectra (MN, Norm–L2, Norm–inf., SNV, MSC and DL).

All models exhibit high accuracy in differentiating between nodes and internodes, even without preprocessing (above 0.7). Notably, ANN exhibited the highest accuracy in predicting and classifying nodes and internodes, particularly in terms of F1-score. LDA and KNN followed, respectively. This baseline performance suggests that the Vis–SWNIR spectral signatures contain inherent discriminatory information. However, preprocessing significantly improves performance, particularly for LDA and ANN, indicating that noise reduction and feature enhancement are crucial for maximizing classification accuracy. KNN, while generally less accurate than ANN, still provides reasonably good performance and offers the advantage of interpretability.

The optimal hyperparameters for each algorithm and preprocessing technique were determined through 5-fold cross-validation. For LDA, the number of components was consistent: one each across all preprocessing techniques (original, MN, Norm–L2, Norm–inf, SNV, MSC, and DL), indicating that minimal dimensionality reduction was required to achieve optimal class separation. For KNN, the optimal number of neighbors varied significantly depending on the preprocessing method, ranging from 4 for MSC to 16 for DL, suggesting that specific preprocessing techniques better preserved the underlying structure of the data. ANN models exhibited more complex interactions between hyperparameters and preprocessing techniques. For instance, with original data, the optimal configuration was identity activation and hidden layer sizes of “128, 128, 128” whereas DL preprocessing, relu activation, and “16, 16” hidden layers performed best. These results highlight the importance of selecting suitable preprocessing methods, which can significantly influence model performance, particularly for non-linear algorithms like ANN.

Compared to previous studies, the results are consistent with findings from [29], which also reported that minimal dimensionality reduction using LDA is effective for spectral data. Similarly, the variation in optimal K values for KNN based on preprocessing aligns with the work of Mancini et al. [30], where the effectiveness of preprocessing in improving KNN performance for spectral data was emphasized. The ANN results corroborate previous research [31], which demonstrated that deeper networks with more neurons tend to perform better for raw spectral data, but more compact networks can be optimal when advanced preprocessing techniques are applied.

### 3.3. External Validation

**Figure 8:**
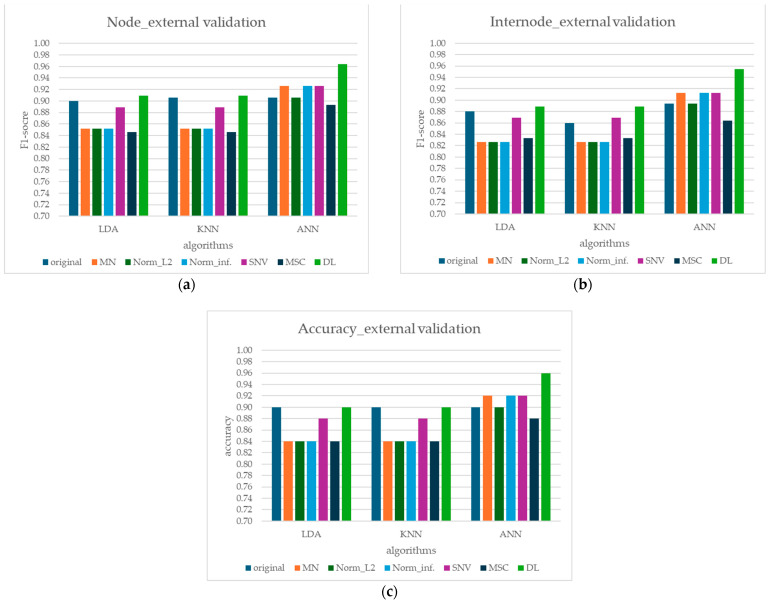
Comparison of performance metrics of external validation models for different preprocessing methods and machine learning algorithms: (**a**) node F1-score, (**b**) internode F1-score, and (**c**) model accuracy.

The performance of the models on an independent set of 50 unknown sugarcane samples further demonstrates their ability to generalize to new, unseen data. The results (Table 4 and Figure 8a–c) show that all models maintain consistent performance metrics compared to those observed during the internal validation test. This consistency suggests that the models have learned meaningful patterns from the spectral data and are not overfitting to the training set. The ANN model yielded the highest average F1-score (>0.90 for all preprocessing methods at the node class and >0.89 at the internode class), suggesting balanced and accurate predictions for both node and internode classes. The choice of preprocessing method significantly impacts model performance. For LDA, DL preprocessing consistently yields the best results, likely due to its ability to enhance subtle spectral differences between nodes and internodes. For ANN, multiple preprocessing techniques, including MSC, Norm_inf, and DL, lead to improved performance, suggesting that the model benefits from various forms of spectral normalization and feature enhancement. KNN’s performance is less sensitive to preprocessing.

## 4. Discussion

This study investigated how to classify the nodes and internodes in stalk sugarcane using Vis–SWNIR spectroscopy in the wavelength range of 400–1000 nm. Several published studies on utilizing NIR spectroscopy combined with ML in the case of sugarcane have been published in the last five years. Among them are those reported for sugarcane disease recognition [19], for predicting sugarcane leaf nutrient content [32], and for monitoring the spatial variability of sugarcane quality in the fields, as reported by Corrêdo et al. [33]. However, this is the first study to use a Vis–NIR spectroscopy descriptor of stalk sugarcane measurements to discriminate between nodes and internodes, combined with a machine learning (ML) algorithm that includes LDA, ANN, and KNN.

In the past, to the best of our knowledge, Vis–SWNIR spectroscopy (400–1000 nm) has not been used for the determination of nodes and internodes in stalk sugarcane classification. However, in the full wavelength range, FT–NIR (1000–2500 nm) has been used to determine sugarcane stalk bending properties characterization and to obtain an RPD value maximum of 4.18 via ANN [34]. This shows that NIR spectroscopy is also effective for evaluating classification cases. This is confirmed by the investigation of the potential of NIR hyperspectral imaging in the spectral range of 930–1630 nm, which was used for the prediction of sugar content in seventy sugarcane stalks of the Khon Kaen 3 variety and obtained a maximum RPD of 1.79 via SVM [35]. This may indicate why Vis–SWNIR, combined with the algorithm of ML, can still perform well because of its ability to utilize variables from Vis–NIR, which are sometimes less informative but can still manage the data to produce useful information.

In Figure 6a, the raw spectra exhibit overlapping features between the two classes, with subtle differences in absorbance intensities across the Vis–SWNIR range. Preprocessing techniques, particularly derivative (DL) transformation, enhance these subtle differences, revealing distinct spectral patterns for nodes and internodes (Figure 6g). The DL spectra highlight variations in the rate of change in absorbance, potentially linked to differences in the chemical composition and physical structure between the two classes. For instance, the prominent peaks around 650–700 nm in the DL spectra, observed more distinctly in internodes, might be associated with chlorophyll or other pigments, which are more abundant in the photosynthetically active internodes compared to nodes [14,36]. The more pronounced peaks and valleys in the derivative spectra of the nodes could be related to their varied textures and the presence of buds, potentially indicating differences in chemical composition or physical structure compared to the smoother internodes [37].

From the calibration model performance results presented in Table 3, it is known that derivative (DL) preprocessing for Vis–SWNIR spectra improves the quality of the spectral signal so that the ML algorithm works better than other types of preprocessing. The ANN model consistently achieves the highest accuracy and F1-scores across all preprocessing methods, suggesting its ability to capture complex, non-linear relationships in the spectral data.

While LDA, being a linear model, exhibits superior performance on the calibration set, achieving perfect accuracy and F1-scores of 1.00, its performance for the validation is not as competitive as ANN or KNN [38]. This discrepancy, along with the higher variability observed in LDA’s performance on the validation and unknown sets, suggests a potential for overfitting. This could be attributed to the limitations of LDA as a linear model in fully capturing the complex, potentially non-linear, relationships present in the spectral data. In contrast, the ANN model, with its ability to model non-linearity, demonstrates more consistent and robust performance across different datasets.

From the external validation set, the results confirm that ANN models, particularly those that incorporate MSC, Norm_inf, and DL preprocessing, exhibit superior performance, further highlighting their robustness and ability to handle diverse spectral data. The performance of LDA on the external validation set is notably lower than that of ANN and KNN, reinforcing the potential limitations of linear models in capturing the complexities of the spectral data for generalization.

Overall, the results of this study highlight the potential of Vis–SWNIR spectroscopy combined with machine learning for rapid and accurate node/internode classification in sugarcane. The superior performance of ANN, especially with appropriate preprocessing, underscores its effectiveness in handling the complexities of spectral data for this classification task. The consistent generalization performance across different models and preprocessing techniques further validates the robustness of the approach. These findings pave the way for the development of automated systems for sugarcane billet preparation and bud chip seedling production, contributing to improved planting efficiency and overall productivity in the sugarcane industry.

## 5. Conclusions

This study successfully demonstrated the potential of Vis–SWNIR spectroscopy coupled with machine learning for rapid and accurate classification of nodes and internodes in the Khon Kaen 3 sugarcane cultivar. Spectra were acquired using a Vis–SWNIR spectrometer within the wavelength range of 400 to 1000 nm. The raw spectra underwent preprocessing using techniques such as MN, Norm_L2, Norm_inf, SNV, DL, and MSC before being fed into classification models. Three algorithms, LDA, KNN, and ANN, were employed to build these models. The results demonstrated that all three algorithms achieved relatively high classification accuracy, with ANN exhibiting the best performance, as evidenced by an average F1-score exceeding 0.90 in all of the calibration, validation and independent datasets. Furthermore, preprocessing significantly enhanced the model’s performance, particularly with methods like SNV, DL, and MSC.

A key contribution of this research is its demonstration of the effectiveness of ANNs in capturing the complex, non-linear relationships present in spectral data for sugarcane node/internode classification. Additionally, the study highlights the importance of spectral preprocessing in improving model performance, with DL preprocessing proving particularly beneficial for both LDA and ANN models. The consistent generalization performance of the models on an independent test set further validates the robustness of the approach.

However, it is important to acknowledge that this study was conducted on a single sugarcane cultivar under controlled laboratory conditions. Further research is needed to evaluate the robustness of the models under varying environmental conditions and across different sugarcane varieties. Additionally, the integration of this technology into a fully automated precision planter requires further development and testing in real-world field settings.

Despite these limitations, this research represents a significant step toward automating key processes in sugarcane cultivation. The findings contribute to the broader field of precision agriculture by demonstrating the potential of NIRS and machine learning for rapid and non-destructive classification tasks, paving the way for more efficient and sustainable crop production systems.

## Figures and Tables

**Table 1:** Hyperparameters and their tuning ranges for the machine learning algorithms.

Algorithm	Hyperparameter	Tuning Range
LDA	Number of components	1–20
KNN	Number of neighbors	1–20
ANN	Hidden layer sizes	(4), (4, 4), (4, 4, 4), (8), (8, 8), (8, 8, 8), (16), (16, 16), (16, 16, 16), (32), (32, 32), (32, 32, 32), (64), (64, 64), (64, 64, 64), (128), (128, 128), (128, 128, 128), (256), (256, 256), (256, 256, 256)
	Activation function	identity, logistic, tanh, relu

**Table 2:** Performance parameters for model evaluation.

Parameter	Meaning	Formula
Accuracy	The overall proportion of correct predictions.	$\frac{TP+TN}{TP+TN+FP+FN}$
Precision	The proportion of positive predictions that were actually correct.	$\frac{TP}{TP+FP}$
Recall	The proportion of actual positives that were correctly identified.	$\frac{TP}{TP+FN}$
F1-score	The harmonic means between precision and recall.	$\frac{2\times Precision\times Recall}{Precision+Recall}$

**Table 3:** Performance of different machine learning models and preprocessing techniques on the calibration and validation datasets.

Dataset	Model	Preprocessing	Internode			Node			Accuracy
			Recall	Precision	F1-Score	Recall	Precision	F1-Score	
Calibration	LDA	Original	1.000	0.994	0.997	0.995	1.000	0.997	0.997
Calibration	LDA	MN	1.000	0.987	0.994	0.990	1.000	0.995	0.994
Calibration	LDA	Norm_L2	1.000	0.987	0.994	0.990	1.000	0.995	0.994
Calibration	LDA	Norm_inf	1.000	0.987	0.994	0.990	1.000	0.995	0.994
Calibration	LDA	SNV	1.000	1.000	1.000	1.000	1.000	1.000	1.000
Calibration	LDA	MSC	0.987	0.975	0.981	0.980	0.990	0.985	0.983
Calibration	LDA	DL	1.000	1.000	1.000	1.000	1.000	1.000	1.000
Calibration	KNN	Original	0.936	0.816	0.872	0.835	0.944	0.886	0.879
Calibration	KNN	MN	0.955	0.866	0.909	0.885	0.962	0.922	0.916
Calibration	KNN	Norm_L2	0.955	0.866	0.909	0.885	0.962	0.922	0.916
Calibration	KNN	Norm_inf	0.955	0.866	0.909	0.885	0.962	0.922	0.916
Calibration	KNN	SNV	0.955	0.828	0.887	0.845	0.960	0.899	0.893
Calibration	KNN	MSC	0.981	0.832	0.900	0.845	0.983	0.909	0.904
Calibration	KNN	DL	0.981	0.836	0.903	0.850	0.983	0.912	0.907
Calibration	ANN	Original	0.942	0.850	0.894	0.870	0.951	0.909	0.902
Calibration	ANN	MN	1.000	0.821	0.902	0.830	1.000	0.907	0.904
Calibration	ANN	Norm_L2	0.936	0.864	0.898	0.885	0.947	0.915	0.907
Calibration	ANN	Norm_inf	0.962	0.872	0.915	0.890	0.967	0.927	0.921
Calibration	ANN	SNV	0.974	0.859	0.913	0.875	0.978	0.923	0.919
Calibration	ANN	MSC	0.974	0.869	0.918	0.885	0.978	0.929	0.924
Calibration	ANN	DL	1.000	0.872	0.931	0.885	1.000	0.939	0.935
Validation	LDA	Original	0.837	0.783	0.809	0.783	0.837	0.809	0.809
Validation	LDA	MN	0.791	0.829	0.810	0.848	0.813	0.830	0.820
Validation	LDA	Norm_L2	0.791	0.829	0.810	0.848	0.813	0.830	0.820
Validation	LDA	Norm_inf	0.791	0.829	0.810	0.848	0.813	0.830	0.820
Validation	LDA	SNV	0.837	0.800	0.818	0.804	0.841	0.822	0.820
Validation	LDA	MSC	0.791	0.723	0.756	0.717	0.786	0.750	0.753
Validation	LDA	DL	0.884	0.826	0.854	0.826	0.884	0.854	0.854
Validation	KNN	Original	0.907	0.765	0.830	0.739	0.895	0.810	0.820
Validation	KNN	MN	0.884	0.792	0.835	0.783	0.878	0.828	0.831
Validation	KNN	Norm_L2	0.884	0.792	0.835	0.783	0.878	0.828	0.831
Validation	KNN	Norm_inf	0.884	0.792	0.835	0.783	0.878	0.828	0.831
Validation	KNN	SNV	0.884	0.745	0.809	0.717	0.868	0.786	0.798
Validation	KNN	MSC	0.977	0.808	0.884	0.783	0.973	0.867	0.876
Validation	KNN	DL	0.953	0.804	0.872	0.783	0.947	0.857	0.865
Validation	ANN	Original	0.907	0.796	0.848	0.783	0.900	0.837	0.843
Validation	ANN	MN	0.977	0.808	0.884	0.783	0.973	0.867	0.876
Validation	ANN	Norm_L2	0.907	0.796	0.848	0.783	0.900	0.837	0.843
validation	ANN	Norm_inf	0.907	0.796	0.848	0.783	0.900	0.837	0.843
validation	ANN	SNV	0.930	0.800	0.860	0.783	0.923	0.847	0.854
validation	ANN	MSC	0.930	0.800	0.860	0.783	0.923	0.847	0.854
validation	ANN	DL	0.953	0.804	0.872	0.783	0.947	0.857	0.865

**Table 4:** Generalization performance of the models on an independent test set.

Model	Preprocessing	Internode			Node			Accuracy
		Recall	Precision	F1-Score	Recall	Precision	F1-Score	
LDA	Original	0.833	0.952	0.880	0.962	0.862	0.900	0.900
LDA	MN	0.760	0.905	0.826	0.920	0.793	0.852	0.840
LDA	Norm_L2	0.760	0.905	0.826	0.920	0.793	0.852	0.840
LDA	Norm_inf	0.760	0.905	0.826	0.920	0.793	0.852	0.840
LDA	SNV	0.800	0.952	0.870	0.960	0.828	0.889	0.880
LDA	MSC	0.741	0.952	0.833	0.957	0.759	0.846	0.840
LDA	DL	0.833	0.952	0.889	0.962	0.862	0.909	0.900
KNN	Original	0.808	1.000	0.860	1.000	0.828	0.906	0.900
KNN	MN	0.760	0.905	0.826	0.920	0.793	0.852	0.840
KNN	Norm_L2	0.760	0.905	0.826	0.920	0.793	0.852	0.840
KNN	Norm_inf	0.760	0.905	0.826	0.920	0.793	0.852	0.840
KNN	SNV	0.800	0.952	0.870	0.960	0.828	0.889	0.880
KNN	MSC	0.741	0.952	0.833	0.957	0.759	0.846	0.840
KNN	DL	0.833	0.952	0.889	0.962	0.862	0.909	0.900
ANN	Original	0.808	1.000	0.894	1.000	0.828	0.906	0.900
ANN	MN	0.840	1.000	0.913	1.000	0.862	0.926	0.920
ANN	Norm_L2	0.808	1.000	0.894	1.000	0.828	0.906	0.900
ANN	Norm_inf	0.840	1.000	0.913	1.000	0.862	0.926	0.920
ANN	SNV	0.840	1.000	0.913	1.000	0.862	0.926	0.920
ANN	MSC	0.826	0.905	0.864	0.926	0.862	0.893	0.880
ANN	DL	0.913	1.000	0.955	1.000	0.931	0.964	0.960

## Data Availability

Data are contained within the article.
